# Fitting Potential Energy Surfaces by Learning the Charge Density Matrix with Permutationally Invariant Polynomials

**DOI:** 10.1021/acs.jctc.3c00586

**Published:** 2023-08-10

**Authors:** Younos Hashem, Katheryn Foust, Martina Kaledin, Alexey L. Kaledin

**Affiliations:** † Department of Chemistry & Biochemistry, Kennesaw State University, 370 Paulding Ave NW, Box # 1203, Kennesaw 30144, Georgia; ‡ Cherry L. Emerson Center for Scientific Computation and Department of Chemistry, Emory University, 1515 Dickey Drive, Atlanta 30322, Georgia

## Abstract

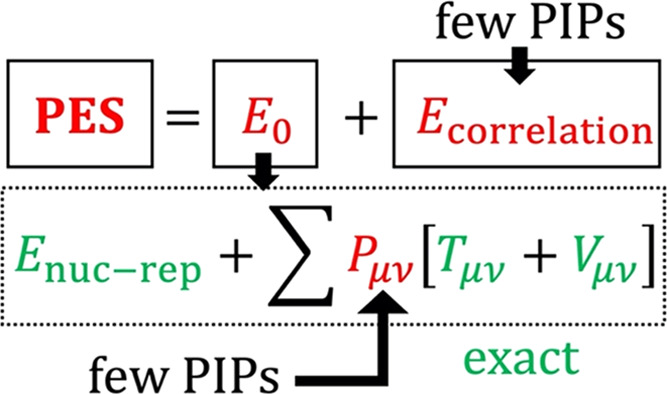

The electronic energy in the Hartree–Fock (HF) theory is the trace of the product of the charge density matrix (CDM) with the one-electron and two-electron matrices represented in an atomic orbital basis, where the two-electron matrix is also a function of the same CDM. In this work, we examine a formalism of analytic representation of a generic molecular potential energy surface (PES) as a sum of a linearly parameterized HF and a correction term, the latter formally representing the electron correlation energy, also linearly parameterized, by expressing the elements of CDM using permutationally invariant polynomials (PIPs). We show on a variety of numerical examples, ranging from exemplary two-electron systems HeH^+^ and H_3_^+^ to the more challenging cases of methanium (CH_5_^+^) fragmentation and high-energy tautomerization of formamide to formimidic acid that such a formulation requires significantly fewer, 10–20% of PIPs, to accomplish the same accuracy of the fit as the conventional representation at practically the same computational cost.

## 1 Introduction

The exciting progress in the analytic representation of molecular interaction potentials commonly known as potential energy surfaces (PESs) and temporally spanning the previous two decades provides a strong stimulus for further advancement of the existing computational methodologies. From the numerous machine learning approaches^1−5^ to variations of the permutationally invariant polynomial theory (PIP) methods,^6−10^ there have been a plethora of unique theories for the description of intermolecular interaction potentials.^11−18^ Notably, several exemplary calculations reported in recent years while using high levels of electronic structure theory have displayed the applicability of the PIP and other similar approaches in a number of such diverse studies as those involving quantum scattering calculations of hydrogen atom abstraction by the OH radical^3^ and reaction dynamics simulations of ionic and radical species on PESs fitted to *ab initio* data^19,20^ and impressively with the inclusion of relativistic effects.^21^ Those and other recently reported calculations have shown, for instance, that to achieve the quality of a PES fit approaching that of the CCSD(T) level of theory, being generally recognized as the “gold standard” in quantum chemistry,^7,9^ one only needs a fraction of training set configurations calculated at this level to make the production of such high-quality PESs practical even for relatively large polyatomic molecules.^9,22,23^

Admittedly, the PIP approach is noteworthy to the present work, given our contemporaneous aims at studying vibrational (infrared and Raman) spectroscopy of molecules, ions, and radicals based on first principles molecular dynamics (MD), including the effects of like nucleus indistinguishability.^24^ Following our recent efforts on large-scale direct MD simulations of molecular vibrational spectra,^25^ we are additionally motivated to apply the PIP approach to larger polyatomic systems since it is a natural way of representing the potential energy, given that permutations of identical nuclei leave the energy unchanged. Furthermore, the method of PIPs is straightforward to apply to essentially any molecular property, such as multipole moment surfaces: dipole moment (DMS), quadrupole moment (QMS), and octupole moment (OMS) surface being the most important ones, and also the polarizability tensor surfaces (PTS), as demonstrated by us previously.^26−28^

In the PIP approach, however, one is unfortunately plagued by the fundamental bottleneck of the permutational symmetry space. That is, to construct a PIP basis of any order for a generic A*_N_*B_*M*_··· molecular system (*N*, *M*,··· being the numbers of atoms of each kind), one must apply a symmetrization operation *N*!*M*!··· times to a generating polynomial while doing so in an (*N* + *M*+···)(*N* + *M*+···–1)/2 vector space of the internuclear distances.^6,7^ The factorial scaling is obviously problematic and has been treated occasionally by simply reducing the full permutational symmetry, as described in some recent works on H_2_–H_2_O PESs.^29,30^

To overcome this obstacle in a more systematic way, some rather ingenious machine learning-based approaches have been extensively explored, from fundamentally invariant PIP theories to a backward differentiation method for PES energies and gradients.^8−10,23,31^ Moreover, the recently reported embedded-atom neural network (NN) calculations examined the possibility of expressing the total energy by breaking it down in Cartesian tensor components of the density, invoking the formalism of basis set expansion of the wavefunction and doing so with highly encouraging results.^32^ The latter method, to be pointed out, is viewed by us as one that is supposed to alleviate the daunting task of generating a high-order PIP by introducing physically meaningful variables^32^ (with mandatory extra fitting parameters) in the nonlinear NN search fields. Others have attempted to achieve the same goal by using symmetry-adapted NNs.^10,17^ As an aside, we mention that molecular properties, including the total energy, can be written out in terms of the electron density, for instance, in the form of the multipole decomposition analysis exemplified by Stone^33,34^ and Price and co-workers,^35,36^ the energy decomposition analysis of Morokuma,^37^ and others.^38−42^ The same may naturally be done for PIP-based PES representation.

However, we note that further assumptions about the explicit physical form of the PESs being fitted using PIPs or incidentally other functions appear to not have been explored. In the conventional PIP approach, the fitting functions are by default constructed as regular polynomials regardless of the PES’s compositional nature, i.e., electron–nucleus and electron–electron interactions, including the electron’s intrinsic spin symmetry. Alternatively, there are perturbation theory approaches of first constructing a low-level PES (*E*_LL_) using an approximate theory, e.g., Hartree–Fock (HF) or density functional theory (DFT) and fitting with one set of PIPs and then fitting the difference energy relative to a high-level (HL) theory, usually CCSD(T): *E*_HL–LL_ = *E*_HL_ – *E*_LL_ with the same or a different set of PIPs in an effort to bring the composite fit to the HL accuracy. The latter approach is commonly known as Δ-machine learning for PESs and has been quite fruitful for cases of small molecules and larger peptides with relatively low permutational symmetry.^4,5,9,22,43−45^ The idea is based on the supposition that *E*_HL–LL_ is a much slower varying function of nuclear configuration than both the *E*_HL_ and *E*_LL_ energies. Therefore, it is supposed that one requires significantly fewer HL points than LL points and consequently fewer PIP terms, making the composite HL fit only marginally more expensive than the laboriously constructed LL fit. Nevertheless, it should be made clear that one still needs to fit the LL data in the first step of this procedure and do so with an extensive set of PIPs with the underlying computational difficulties.

Bearing this point in mind, we introduce below a modified approach to fitting a PES to a training set of data obtained at a correlated but relatively cheap level of theory, e.g., DFT. The approach is inspired by a combination of the embedded-atom NN and the Δ-machine learning procedures, described above. We implement our approach by assuming that a typical PES has an approximate form of the Hartree–Fock energy, and simultaneously we treat Δ*E*, formally the correlation energy, as a correction energy in the sense of *E*_HL–LL_. We propose to do so with a comparatively low-order PIP by taking advantage of the properties of *E*_LL_ and Δ*E*. If desired, additional corrections to bring the fit to an HL quality (MP2, CCSD, *etc*.) can be made by applying the conventional Δ-machine learning procedure, although we will not consider this step in the present work as it is not essential to the discussion.

We briefly recall that the exact electronic energy in the sense of a full configuration interaction (FCI) in a given basis set may be written as a sum of the Hartree–Fock energy and the correlation energy
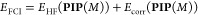
1where **PIP**(*M*) indicates a linear combination of PIPs defining the corresponding energy term of some total order *M*, shared by both terms. For a typical closed-shell system dominated by a single determinant, the correlation energy is a small fraction of *E*_FCI_ (as will be shown in Section 3). From this perspective, eq 1 is homologous with the Δ-machine learning formulation having the following choices: *E*_LL_ = *E*_HF_, *E*_HL_ = *E*_FCI_, and *E*_HL–LL_ = *E*_corr_, the correlation energy. For practical purposes, *E*_FCI_ may be any post-HF or even a DFT level of theory, with optionally added empirical dispersion corrections, with *E*_HL–LL_ representing the correlation energy, a slowly varying function of the nuclear configuration. The point where the present formulation differs in technique from the existing ones is in treating *E*_HF_(**PIP**) as a sum of electron density components fitted individually while treating *E*_corr_(**PIP**) using PIPs in a conventional manner, to be elucidated below. The premise of this approach is that both of the key terms (*E*_HF_ and *E*_corr_) can be fitted simultaneously and with a similar level of precision while using one set of low-order PIPs.

## 2 Computational Methods

In the closed-shell Hartree–Fock theory, the total electronic energy, with the nuclear repulsion energy added, is a sum over the 1- and 2-electron Hamiltonian matrix elements in an AO basis set^46,47^
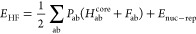
2For formulaic compactness, not dissimilar to the minimal basis DFTB,^48^ we assume a single primitive basis function per atom center *a* and a generic basis set {ϕ_a_}_*K*_; extension to larger basis sets is straightforward. In the above, **P** is the (bond order) charge density matrix defined as a sum of density matrices over the occupied orbitals *I* with the MO coefficients *d*_Ia_, namely, *P*_ab_ = 2∑_*I*_*d*_Ia_^*^d_Ib_, although, for the present purposes, its formal definition is unimportant. **H**^core^ is the core Hamiltonian matrix, a one-electron operator embedding the electron kinetic energy and the Coulomb interactions of the electrons with the nuclei
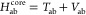
3aso formally, one has^47^
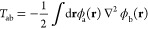
3b
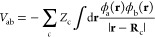
3cto be integrated over the electron coordinates **r** while **R**_c_ is the position of atom c with nuclear charge *Z*_c_. Finally, **F** is the Fock matrix consisting of the one-electron core Hamiltonian and a two-electron contribution, **G**
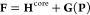
4a
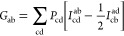
4b

The quantities inside the square brackets in eq 4b are the two-electron integrals
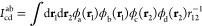
5

If we use a Cartesian Gaussian basis set, i.e.,
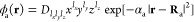
6where *D*_*l*_*x*_*l*_*y*_*l*_*z*__ is the normalization constant and *l*_*x*_*l*_*y*_*l*_*z*_ are the orbital quantum number integers, then eqs 3–5 can be expressed analytically in terms of Gaussian and error functions with the α_a_ exponents as parameters (see the Supporting Information for details).

Rewriting eq 2 in an expanded form shows in a clearer way how the HF energy can be parameterized in terms of the elements of **P**
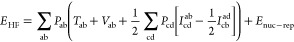
7since all of the quantities inside the brackets are parameterized functions of α_a_ and **R**_a_.

To continue, we express the diagonal density matrix elements *P*_aa_ using PIPs as we would express the partial charges in a multipole representation^27,28^
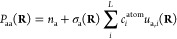
8where *n*_a_ is the permanent electron population on atom a (e.g., *n*_a_ = 8 for Oxygen); *c*_*i*_^atom^ are linear expansion coefficients expressing all of the atomic populations in the given atom class; *u*_a*,i*_ is the *i*-th PIP of the total power 0 < *m*_*i*_ ≤ *M* describing atom a, and σ_a_ is a long-range damping function (see the SI for exact definitions). Here, we use the collective Cartesian coordinate **R** to describe a nuclear configuration. The PIPs are expressed in the usual *N*(*N* – 1)/2 internuclear distances employing a permutationally symmetrized sum of monomial products by cycling over all possible permutations *J*
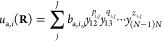
9and symmetrized with the binary phase factors *b*_a*,i,j*_ = ±1.^28^ The phases ensure covariance of *u*_a*,i*_(**R**), meaning that a permutation of two identical nuclei, e.g., 1 and 2, results in a corresponding exchange of *P*_11_ and *P*_22_ so that the total energy is invariant under the permutation. For the atom pair (a,a′), the usual choice for the internuclear distance function is a Morse variable, *y*_aa′_^*p*_*i,j*_^ = exp(−*p*_*i,j*_*d*_aa′_/*d*_0_), where *p*_*i,j*_ is an integer power, *d*_aa′_ = |**R**_a_ – **R**_a′_|, and *d*_0_ is a parameter. The integer powers satisfy the relation *p*_*i,j*_ + *q*_*i,j*_ + ··· + *z*_*i,j*_ = *m*_*i*_. All combinations of a given integer set, which satisfy the like atom permutational symmetry rules, are considered for each *m*_*i*_. For a chosen maximal polynomial power *M*, this determines the PIP basis size *L*.

The off-diagonal elements, specifically those connecting two different atom sites, are expressed similarly and with a short-range damping factor to ensure a proper wavefunction behavior at the united atom limit
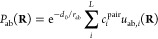
10but using differently symmetrized PIPs. For instance, a permutation of like nuclei 1 and 2 must result in *P*_12_ ↔ *P*_21_ and simultaneously in *P*_1*c*_ ↔ *P*_2*c*_ for *c* = 3,···, *N*. This is enforced by constructing pair-polynomials using direct products of the single phases^28^
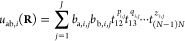
11where we have incidentally used an independently scaled set of the internuclear distance functions, namely, *t*_aa′_^*p*_*i,j*_^ = exp(−*p*_*i,j*_*d*_aa′_/*t*_0_), with *t*_0_ being another range parameter. The coefficients *c*_*i*_^atom^ and *c*_*i*_^pair^ (for *i* = 1, *L*) of each of the symmetry-unique species are determined by formulating and solving a least-squares problem. The density matrix elements must, in principle, satisfy the conservation of charge, or the total number of electrons, *n* = ∑_ab_*P*_ab_*S*_ab_, where the orbital overlap is *S*_ab_ = ∫d**r**ϕ_a_(**r**)ϕ_b_(**r**). This condition may be enforced simultaneously with the main procedure of PES fitting but with a properly chosen least-squares weight.

As one may expect, the form of eq 7 does not allow for a linear least-squares solution for the expansion coefficients due to the Fock matrix dependence on the density. At present, we wish to consider only a linear class of solutions, due to its simplicity of application by a linear regression analysis. Therefore, we have examined several cases of linear approximations to eq 7 by manipulating elements of the 2-electron matrix **G**; however, in the calculations reported below, we describe in detail the simplest and most effective approximation, namely, the **G** = 0, or the core Hamiltonian model (CHM). In the course of numerous tests, we have found that the other linear models considered here yielded essentially the same results as the CHM model. (Details of those models and corresponding test calculations are summarized in Table S1 of the SI.)

Now, substituting eq 7 into 1 and assuming the CHM model, we obtain a fitting equation for a generic PES given by *ab initio* data on the left-hand side
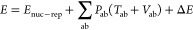
12and a linearly parameterized function on the right-hand side, with the elements *P*_ab_ expressed using eqs 8 and 10, and the correction energy parameterized in the usual way
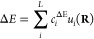
13with the definition of *u*_*i*_(**R**) = ∑_*j*_^*J*^*y*_12_^*p*_*i,j*_^*y*_13_^*q*_*i,j*_^···*y*_(*N*–1)*N*_^*z*_*i,j*_^. The number of linear coefficients in eq 12 depends on the size of the orbital basis in eq 6 and the number of different atom types, with the exact dimensions specified for each particular system in the calculations below. The coefficients are found by linear regression using the QR decomposition^49^ as implemented in the DGELS subroutine contained in the MKL software suite.

We also note that there still remain nonlinear parameters in eqs 12 and 13, namely, the range parameters *d*_0_, *t*_0_, and the Gaussian exponents α_a_ for each atom type appearing in eq 6. Exploratory calculations have indicated so far that varying these parameters on a judiciously chosen range can significantly reduce the overall root-mean-square error (RMSE). Thus, all of the results reported below correspond to the variationally best set of these exponential parameters. Additionally, we presently limit ourselves to the *s*-electron density so as to minimize the overall number of fitting variables, linear and nonlinear, and consequently, the amount of *ab initio* data while demonstrating the proof of principle using the simplest model possible. In fact, the *s*-density approach was found to produce excellent results for dipole, quadrupole, and octupole fits of polyatomic molecules using PIPs,^28^ while extension to *p*, *d*, ··· electron density is formally straightforward and will be examined in future publications. Incidentally, in the discussion below, we make the following additional definitions: the conventional *M*-th order PIP representation of a PES is referred to as PIP(*M*), while the core Hamiltonian model (eq 12) is called CHM(*M*).

All electronic structure calculations carried out to produce training and testing set data sets were performed with Gaussian^50^ and MOLPRO^51^ suites of codes.

## 3 Results and Discussion

### 3.1 Two-Electron Systems: HeH^+^ and H_3_^+^

**Figure 1:**
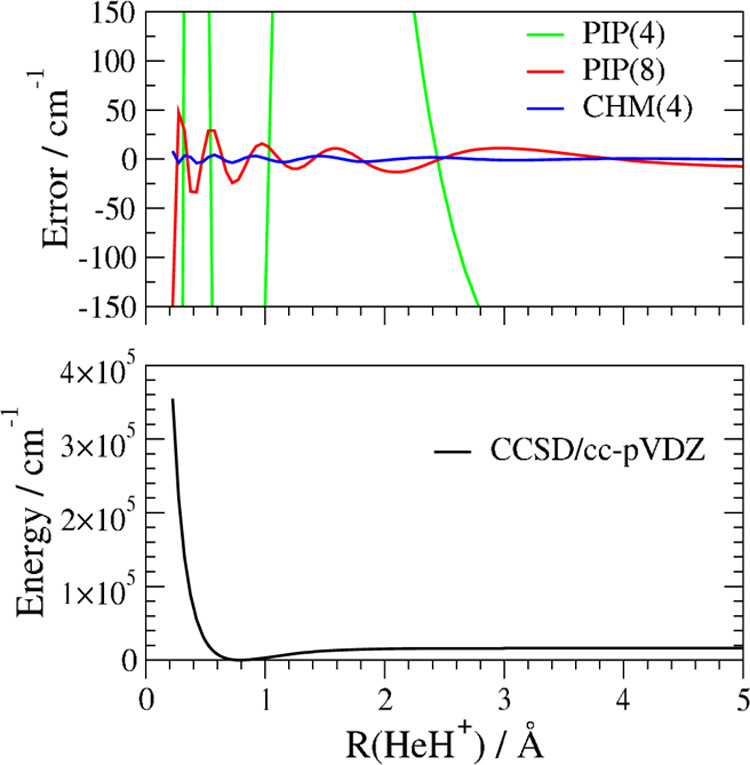
Test set errors (the upper panel) of three representative fits of the HeH^+^ potential: PIP(4) and PIP(8) are the conventional PIP representations of the 4 and 8 order, respectively, and CHM(4), the core Hamiltonian model, is represented with a PIP order of *M* = 4. The *ab initio* test data curve is shown in the lower panel where the zero of energy is the equilibrium distance *R* = 0.79 Å.

**Figure 2:**
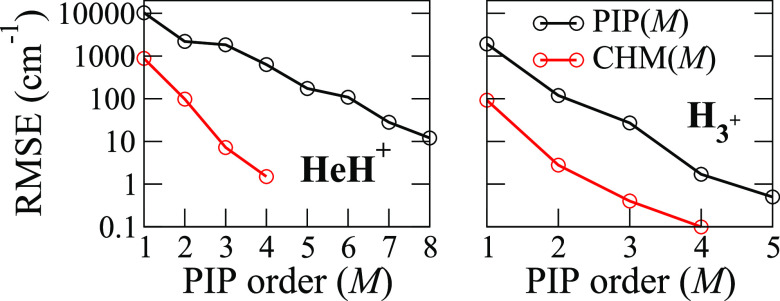
Comparison of the RMSE of the HeH^+^ and H_3_^+^ fits of their potential energies calculated at the CCSD/cc-pVDZ level of theory and plotted on the log_10_ scale versus the PIP order *M*. 100 and 3000 points were used for HeH^+^ and H_3_^+^, respectively.

An informative point to start is to investigate CHM on a two-electron system since the correlation energy can be calculated exactly for a given basis set, and the approximation of the CHM model to the exact HF energy is less drastic than for a multielectron system. We first consider a diatomic case, HeH^+^, for which we calculate the potential curve at the CCSD/cc-pVDZ level and generate 100 points for training the model and another 100 points, situated at the midpoints of the training set, for testing the fit. For the CHM model, we use a single Gaussian *s*-type function for He and H atoms to represent the electron density and assign the following permanent atomic populations: *n*_He_ = 2, *n*_H_ = 0. The number of linear coefficients in the CHM model is 4*L*, which includes *L* terms for each of *P*_He_, *P*_H_, *P*_HeH_, and the correction energy Δ*E*. The results of these calculations are presented in Figures 1 and 2.

We first point out that the potential energy curve spans an extremely broad range of ∼360,000 cm^–1^ (∼44.6 eV) at the repulsive wall with *R* = 0.225 Å to 0 at the equilibrium with *R* = 0.79 Å, as seen in Figure 1; the binding energy to the He + H^+^ products is 16,094 cm^–1^. Second, a comparison of RMSE convergence shows that the CHM model attains a ∼1 cm^–1^ precision with *M* = 4, while the conventional PIP model with *M* = 4 shows an error of ∼1000 cm^–1^ and is still at ∼10 cm^–1^ with the 8th order polynomial, as can be seen in Figure 1. More importantly, the distribution of the error for the CHM model is relatively uniform and mildly oscillatory at the repulsive wall, with the maximum value of 8 cm^–1^ occurring at *R* = 0.225 Å. The PIP(8) and especially PIP(4) models execute large oscillations at the values of *R* shorter than the equilibrium, suggesting perhaps that treatment of the repulsive wall, which also includes the nuclear repulsion term, is somewhat problematic with a bare exponential function. Others have observed similar behavior in the fitting of high-energy regions of methanium ion and used appropriately modified internuclear distance functions.^52^ Energy decomposition of the CHM(4) fit, provided in Figure S1 of the SI, shows a dominant contribution of the Hartree–Fock component, which does include the nuclear repulsion energy, while the Δ*E* term behaves as correction on the [0.4, 1.0] Å region that roughly encompasses the vicinity of the equilibrium state.

Another illustrative case is the 3-center/2-electron H_3_^+^ ion, which may be regarded as a product of splitting the He atom into an H_2_ (*D*_2_ if the mass were to be conserved) molecule to create two additional vibrational degrees of freedom and, therefore, 2- and 3-body coupling terms exemplified by *y*_12_*y*_13_ and *y*_12_*y*_13_*y*_23_ and various mixed powers. For this ion, we also generated a test set using the CCSD/cc-pVDZ level of theory evaluated along an NVE trajectory with the total energy approximately equal to the harmonic ZPVE and one quantum of the asymmetric stretch (7335 cm^–1^). To reinforce the point made in Section 1, we show in the SI (Figure S2) that the correlation energy is indeed a much more slowly varying function of the nuclear configuration than the total energy. A single Gaussian *s*-function was placed on each H center, resulting in 3*L* linear terms (*P*_H_, *P*_HH_, and Δ*E*) while setting *n*_H_ = 2/3 for the formal total charge of +1. We used 3000 points to generate a series of fits, summarized in Figure 2. A pattern that one observes in the RMSE plots is that the CHM model tends to “run ahead” of the conventional PIP model by two powers of the total PIP order *M*, namely, the same accuracy is achieved by CHM(*M* – 2) as by PIP(*M*).

What is also significant is that an augmented CHM(1) model can perform comparably to a complete PIP(4) level of treatment. Calculations show that the inclusion of the one-body *y*_*ij*_^*p*^ and *t*_*ij*_^*p*^ terms with *p* = 2, 3, and 4 into the PIP basis of the CHM(1) model produces RMSEs of 15, 2, and 1.6 cm^–1^, respectively. The latter value is to be compared with the PIP(4) RMSE of 1.7 cm^–1^. Incidentally, augmenting the same basis for PIP(1) with the aforementioned one-body terms does not lead to a better performance than the full PIP(2). These findings may be explained by the fact that PIP(1) is, by definition, missing 2-body terms, while CHM(1) does indeed contain such terms through the *P*_ab_*H*_ab_^core^ products. (Full details of these fits can be found in Table S2.)

**Figure 3:**
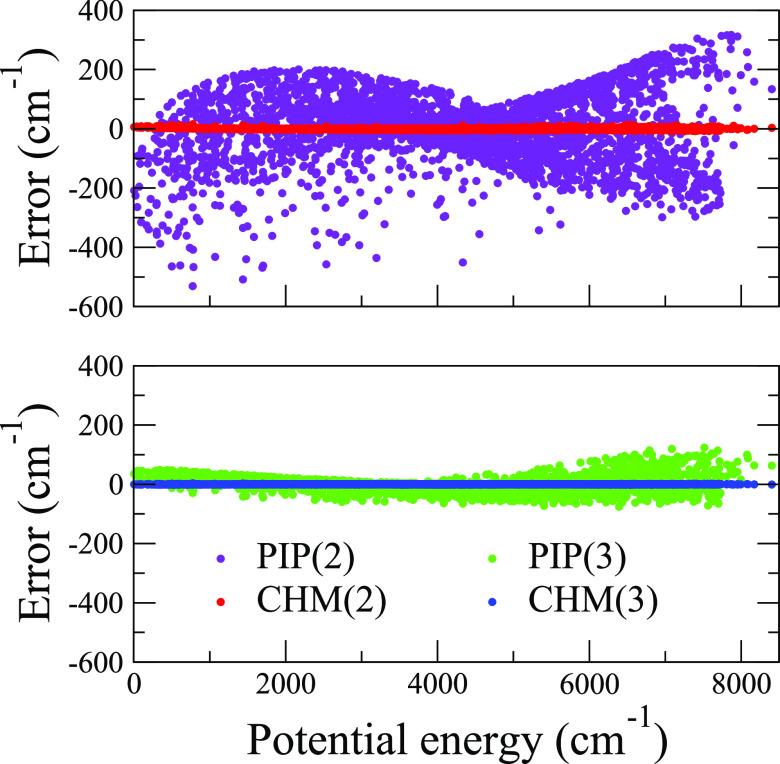
PIP(*M*) and CHM(*M*) training set errors for PES fits to H_3_^+^ CCSD/cc-pVDZ potential energy data, with the horizontal axis being relative to the global energy minimum. The upper and lower panels compare *M* = 2 and *M* = 3 fits, respectively. One observes an essentially complete convergence of the CHM model at the PIP order of *M* = 2.

To further illustrate the robustness of CHM, we compare side-by-side the errors for *M* = 2 and *M* = 3 fit levels as functions of the potential energy above the global minimum, as shown in Figure 3. One can see that PIP(2) fails rather dramatically across the entire energy range while CHM(2) produces a remarkably close fit with RMSE = 2.8 cm^–1^. PIP(3) is a major improvement up to 4000 cm^–1^ above the minimum, beyond which the error still oscillates by more than 100 cm^–1^. CHM(3) is essentially converged at this level with the RMSE = 0.4 cm^–1^. In other words, as the above results indicate, the addition of a third center to a 2-center/2-electron system (HeH^+^) is described impressively well by CHM using a low-order PIP basis.

### 3.2 Methanium Fragmentation Dynamics: CH_5_^+^ → CH_3_^+^ + H_2_ → CH_4_ + H^+^

One of the first examples of the use of PIPs in a global PES fitting was reported for CH_5_^+^,^52^ a system with highly fluxional hydrogens where enforcing permutational symmetry is crucial for correctly describing its dynamical properties. Therefore, methanium is an excellent case for the present methodology. The existing conventional PIP-derived PES^52^ has been an important benchmark used in a number of applications, and our present aim is to demonstrate the performance of the CHM model relative to its conventional PIP counterpart. For rigorous testing of the model, we consider three configurational basins: (I) a vibrationally hot but intact CH_5_^+^; (II) a short-range and long-range interaction of the H_2_ and CH_3_^+^ moieties; and (III) a small amount of the CH_4_ + H^+^ fragments configurations.

For basin-I, we propagate an NVE trajectory with the total energy of 16,000 cm^–1^ for 50,000 steps and a 1 fs time integration step. Every 5th point was included in the training set. For basin-II, we carried out three sets of biased NVE simulations, (A) by adding a harmonic potential connecting C and H1 with the C–H1 equilibrium distance 2 Å and the force constant of 0.5 hartree/bohr^2^ setting the initial kinetic energy of 5000 cm^–1^ and propagating the trajectory for 4000 time steps with Δ*t* = 1 fs. Every 4th point was included in the training set; (B) same as set-A but with the bias potential centered at C–H1 = 3 Å, and every 4th point included in the training set; (C) same as set-B but with a second added potential connecting C and H2 at the 3 Å equilibrium distance and every 5th point included in the training set. To generate points for basin-III, we repeated the calculation for basin-II/set-B but with the C–H1 bias potential distance extended to 4 Å, saving every 5th point of the 4000 steps trajectory into the training set. In total, 13,605 training set points were generated. The remaining points not included in the training set (52,400 in total) were saved for testing the fits. These calculations were carried out at the B3LYP/cc-pVTZ level of theory.

**Figure 4:**
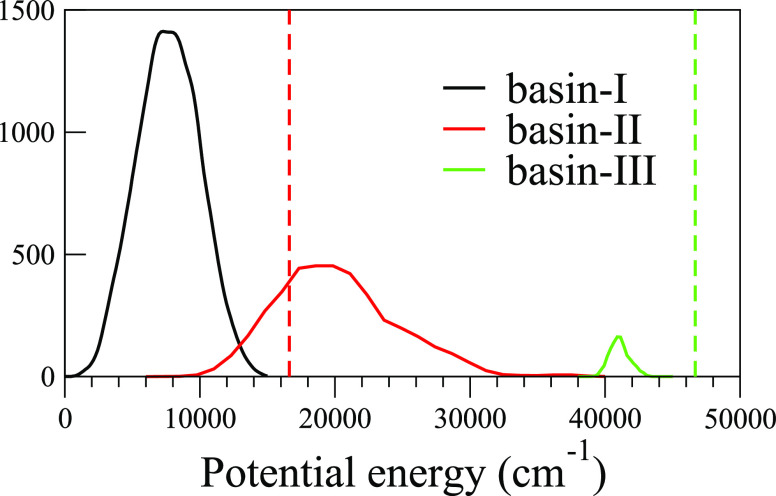
Distribution of the potential energy of CH_5_^+^ relative to the global minimum, calculated at the B3LYP/cc-pVTZ level of theory. The three basins, as defined in the text, are the (I) vibrationally hot CH_5_^+^, and the (II) CH_3_^+^/H_2_ and (III) CH_4_/H^+^ regions. The dashed vertical lines indicate the CH_3_^+^ + H_2_ (red) and CH_4_ + H^+^ (green) dissociation limit asymptotes.

As seen in Figure 4, basin-I and basin-II make up most of the training set space, with basin-I extending from the vicinity of the global minimum to 14,000 cm^–1^ (∼1.3 ZPVE at the harmonic level) with at least one vibrational quantum of any one of the 12 normal modes excited. The much broader basin-II spans from 10,000 to 32,000 cm^–1^ range overlapping with basin-I just below the CH_3_^+^ + H_2_ dissociation asymptote. Finally, the much narrower basin-III is centered around 41,000 cm^–1^ and reflects a low vibrational energy CH_4_ interacting with the proton that orbits it at a roughly 4 Å C–H^+^ radius. (Pair radial distribution functions are provided in Figure S3 of the SI.)

**Figure 5:**
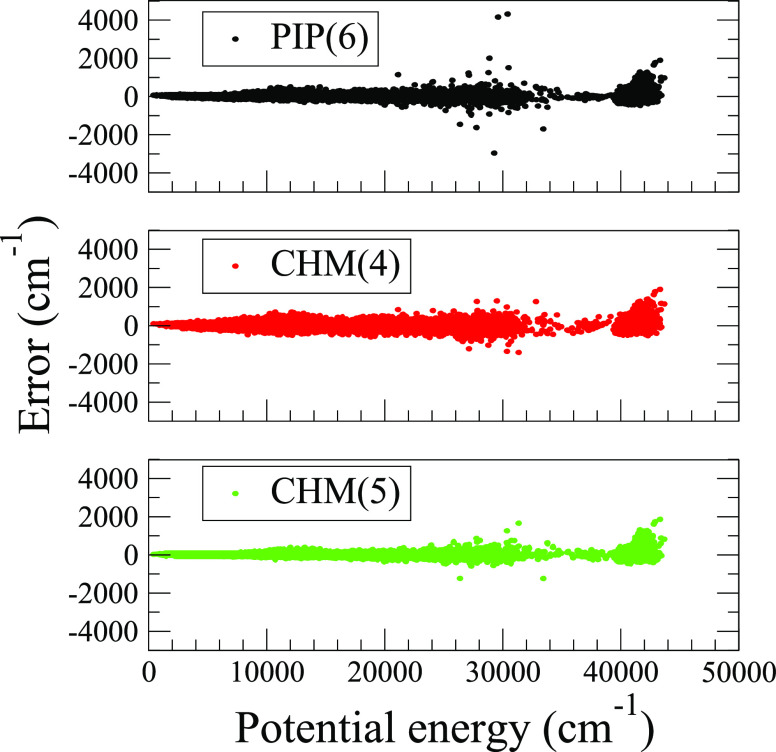
PES test set errors of CH_5_^+^ fits trained on 13,605 configurations and tested on 52,400 configurations, calculated at the B3LYP/cc-pVTZ level of theory, and shown here for three models: PIP(6), CHM(4), and CHM(5). The potential energy is relative to the global minimum. The respective training set RMSEs are 73, 115, and 58 cm^–1^, and the corresponding testing set RMSEs are 87, 118, and 68 cm^–1^.

Following the generation of the sets, we trained the models on the 13,605 point set (cf. Figure 4) for PIP orders *M* = 1–6, with the respective PIP basis sizes *L* = 3, 10, 32, 101, 299, and 849. For the CHM model, the linear spaces are *L*′ = 5 (*L* – 1) + 1 = 11, 46, 156, 501, 1491, and 4241 due to the elements *P*_H_, *P*_C_, *P*_HH_, *P*_CH_, and Δ*E*. The permanent electron populations were set to *n*_C_ = 6 and *n*_H_ = 4/5 so as to formally define the total charge to be +1. Fitting weights of 1 and 0.01 were used for the potential energy *E*(**R**) and the configuration-dependent electron population *n*(**R**) = ∑_ab_*P*_ab_(**R**)*S*_ab_(**R**), respectively. The small weight for the latter was introduced to prevent the individual density elements from an occasional unphysical oscillation at short internuclear distances. We then tested the fits on the 52,400-point set. A complete summary of the fits may be found in the SI Table S3, while in Figure 5, we examine the test error of the best conventional fit, PIP(6) with test RMSE = 87 cm^–1^, against two lower order fits CHM(4) with test RMSE = 118 cm^–1^ and CHM(5) with test RMSE = 68 cm^–1^. First, we note that the RMSEs, both for the training and test sets, are comparable, with CHM(4) performing slightly worse than PIP(6) and CHM(5) performing slightly better. For all three fits, the errors on the test set tend to increase slowly and monotonically, encompassing basins-I and II, up to ∼30,000 cm^–1^, the energy where the PIP(6) model exhibits several outliers, which are not readily identifiable as outliers in the CHM models. Five such structures, which we loosely assigned as H_2_CH–H–H “giraffe”-like motifs (see Figure S4 for illustrations), are found in the test set, with only one closely related structure found in the training set, reflecting the test-to-train point ratio. The largest test errors for these five structures are 4317 cm^–1^ for PIP(6), 454 cm^–1^ for CHM(4), and 628 cm^–1^ for CHM(5), suggesting that the CHM model is better behaved, i. e. more reliable. At the top of the energy range near 40,000 cm^–1^, the test errors in basin-III, on which the models were trained with only 800 points (∼5% share of the full training set), tend to exceed those of basins-I and II slightly and are fairly similar for the three levels of treatment.

Summarizing this section, we emphasize that only 101 PIPs with 501 linear parameters were required for the CHM model to approach the accuracy of PIP(6) having 849 PIPs with as many linear parameters, and 299 PIPs with 1491 linear parameters were sufficient for CHM to outperform the latter. In other words, the ratios of linear space dimensions 501/849 = 0.6 and 1491/849 = 1.7 suggest that the CHM(4) and CHM(5) models do not require significantly more parameters and thus *ab initio* training data than PIP(6) for a fit of a comparable RMSE with a similar level of fidelity. Furthermore, as can be seen in Table 1, the corresponding CPU times to calculate the potential are 1200 μsec for PIP(6) and 1100 and 4100 μsec for CHM(4) and CHM(5), respectively, exposing the same effort for the CHM(4) model and a factor of ∼3 increase in computational complexity of the CHM(5) model. The above points are the most obvious and important practical properties of the CHM formulation we wish to communicate at the present stage.

**Table 1:** CPU Times in Microseconds for a Single-Point Energy Calculation of Methanium (CH_5_^+^) and Formamide (H_2_NCHO) Using the Two Models with the Various PIP Orders *M*^a^

	CH_5_^+^		H_2_NCHO	
*M*	PIP(*M*)	CHM(*M*)	PIP(*M*)	CHM(*M*)
1	2	32	4	64
2	3	77	6	86
2*			16	110
3	24	330	37	390
3*			100	710
4	110	1100	130	
5	370	4100	580	
6	1200	14,000		

a The two augmented PIP basis sets used for the formamide are marked as 2* and 3*. (The calculations were done on an Intel Xeon 4210 “Cascade Lake-SP” 2.2 GHz node using a single core).

### 3.3 High-Energy Tautomerization of Formamide

As recent reports on the state-of-the-art of fitting the PES of peptides have amply demonstrated,^9^ the need for a high-level electronic structure treatment of such systems is necessary for the accurate description of isomerization energies and proton transfer dynamics, including tunneling, bond breaking/formation, interaction with water, and other processes. Here, we explore application of the CHM method to fitting the PES of formamide, a derivative of formic acid that has the typical signatures of a peptide in the Amide A, I, II, III vibrational frequency range.^53,54^ Formamide possesses two main isomer basins, the global H_2_N–HC=O minimum (**MIN1**) and a HN=CH–OH minimum (**MIN2**) that is 4264 cm^–1^ higher and is formally a formimidic acid based on our calculations at the B3LYP/aug-cc-PVDZ/GD3 level of theory. The tautomerization of formamide into formimidic acid is well studied in the literature, and our results generally agree with past theoretical studies.^55−61^ The two basins are connected by a high-energy proton transfer transition state (**TS12**) 16,503 cm^–1^ (47.2 kcal/mol) above the global minimum (see Table S4 for full energetics). The energy barrier for the formamide/formimidic acid isomerization was calculated by Wang et al.^58^ to be 48.9 kcal/mol in the gas phase. They also showed that the H_2_O-assisted barrier height lowers to 22.6 kcal/mol.^58^**MIN1** can isomerize into another identical structure via NH_2_ rotation over either one of two respective transition states **TS1** and **TS2**, 6342 and 6842 cm^–1^ above **MIN1**. On the other hand, the formimidic acid, **MIN2**, can form other low-energy isomers **MIN3-MIN5** via various NH and OH bond rotation saddle points^54,59^ (see Tables 3 and S4 and Figure S5 for the energy landscape details).

To generate the training set, we propagated five trajectories for each of the two main isomers with the following total energies above their respective minima: 1241, 2482, 4964, 9928, and 19,856 cm^–1^ for the **MIN1** structure and 1258, 2516, 5033, 10,066, and 20,133 cm^–1^ for the proton transfer **MIN2** structure. These trajectories were propagated for 10,000 steps, each with a 1 fs time integration step and from which 5322 points were selected from the **MIN1** batch, and 5489 points were similarly selected from the **MIN2** batch. The method of selection, which is a form of pruning, was described by us elsewhere,^24,28^ with additional details given in the SI. Thus, selected points contain all of the isomers of the **MIN1** and **MIN2** basins. However, they do not describe the high-energy proton transfer saddle point, **TS12**. To represent the latter, we carried out constrained MD simulations by placing harmonic springs on the OH′ and NH′ bonds (H′ is the shared hydrogen) with the spring equilibrium distances corresponding to the **TS12** structure, e.g., 1.337 and 1.343 Å. One short simulation was done with stiff force constants (0.2 hartree/bohr^2^) and low initial kinetic energy (1000 cm^–1^) for 1000 steps so as to sample the region of the close vicinity of **TS12**. Another longer simulation was propagated with looser force constants (0.1 hartree/bohr^2^) and a higher initial kinetic energy (5000 cm^–1^) for 10,000 steps to sample configurations farther away from the saddle point. From these constrained trajectories, 333 (every third) and 1428 (every seventh) points were selected for the training set, culminating in a total of 12,572 training points. A testing set of a total of 20,852 points was also prepared using similar selection methods. The above MD simulations were carried out at the level of B3LYP/aug-cc-pVDZ/GD3^62^ using Gaussian-16. At these configurations, we also generated energies at the explicitly correlated PNO-LCCSD(T)-F12/cc-pVDZ-F12^63^ level to investigate whether CHM is sensitive to the level of electron correlation treatment. The latter calculations were done using MOLPRO 2022.2. (Figure S6 shows the potential energy distributions of both sets.)

We used PIPs of order *M* = 1–5 to train the models with the respective basis sizes *L* = 8, 46, 220, 905, and 3292. We also used two augmented bases: *M* = 2* with *L* = 111 containing limited 3rd order terms of the form *y*_*ij*_^3^ and *y*_*ij*_^2^*y*_*kl*_ and *M* = 3* with *L* = 611 containing limited 4th order terms of the form *y*_*ij*_^4^, *y*_*ij*_^3^*y*_*kl*_, *y*_*ij*_^2^*y*_*kl*_^2^, and *y*_*ij*_^2^*y*_*kl*_*y*_*mn*_. Unlike for PIP(*M*), for the CHM(*M*) model, we require a much larger linear coefficient space for the same basis *L* due to the density matrix elements and the standalone Δ*E* term totaling in *L*′ = 12(*L* – 1) + 1 coefficients, with exact numbers listed in Table 2. As in the case of CH_5_^+^, we assigned the weights of 1 for the PES data and 0.01 for the total charge, with the latter weight being used primarily to curb any excessive oscillations in the density elements rather than to closely conserve the total charge value, which is practically irrelevant to the quality of the PES. Examination of the testing set RMSEs, which we summarize in Table 2 (the training set RMSEs are presented in Table S4 of the SI), shows that the CHM errors from the two electronic structure methods are quite similar. The CCSD(T) errors are slightly bigger, and that is understandable since we used the same exponents that were initially optimized for the B3LYP data. We view it as a positive result, and it suggests that the nonlinear parameters inherent in the present formulation may be preoptimized using a low-quality *ab initio* data set and subsequently be used as input for the later fitting of high-quality data.

**Table 2:** Test Set RMSE (in cm^–1^) of H_2_NCHO Fits for the Two Electronic Structure Levels Considered in the Present Work: “B3LYP” = B3LYP/aug-cc-pVDZ/GD3 and “CC” = PNO-LCCSD(T)-F12/cc-pVDZ-F12^a^

			B3LYP		CC	
*M*	*L*	*L*′	PIP(*M*)	CHM(*M*)	PIP(*M*)	CHM(*M*)
1	8	85	4106.4	1278.3	4181.1	1278.6
2	46	541	2073.6	368.0	2064.2	375.5
2*	111	1321	1174.0	156.9	1183.0	160.9
3	220	2629	914.8	72.0	929.7	74.0
3*	611	7321	361.3	39.7	367.6	41.8
4	905	10849	276.3		283.1	
5	3292	39493	60.4		64.8	

a Several PIP orders *M* with the corresponding basis size *L* were considered, where 2* and 3* are the augmented sets (see text). The linear coefficient dimension *L*′ = 12 (*L* – 1) + 1 for the CHM model is also provided.

Second, it is evident that the CHM model converges with *M* (and *L*) much faster than the conventional PIP model, namely, the CHM(3) fit (*L* = 220, *L*′ = 2629) is of comparable quality with PIP(5) (*L* = 3292), and the augmented CHM(3*) fit (*L* = 611, *L*′ = 7321) is of better quality. The ratios of the linear space dimensions, i.e., 2629/3292 = 0.8 and 7321/3292 = 2.2 indicate that the CHM models do not require significantly more parameters, and therefore training data points, than the PIP model to achieve similar or better quality fits. Importantly, the computational costs for a single-point energy calculation are also comparable. Take, for example, the 580 μsec for PIP(5) versus 390 and 710 μsec for CHM(3) and CHM(3*), respectively (cf. Table 1). We also note that the CHM fits appear to be trained relatively well, given that the testing set RMSEs are not significantly greater than the corresponding testing set RMSEs.

**Figure 6:**
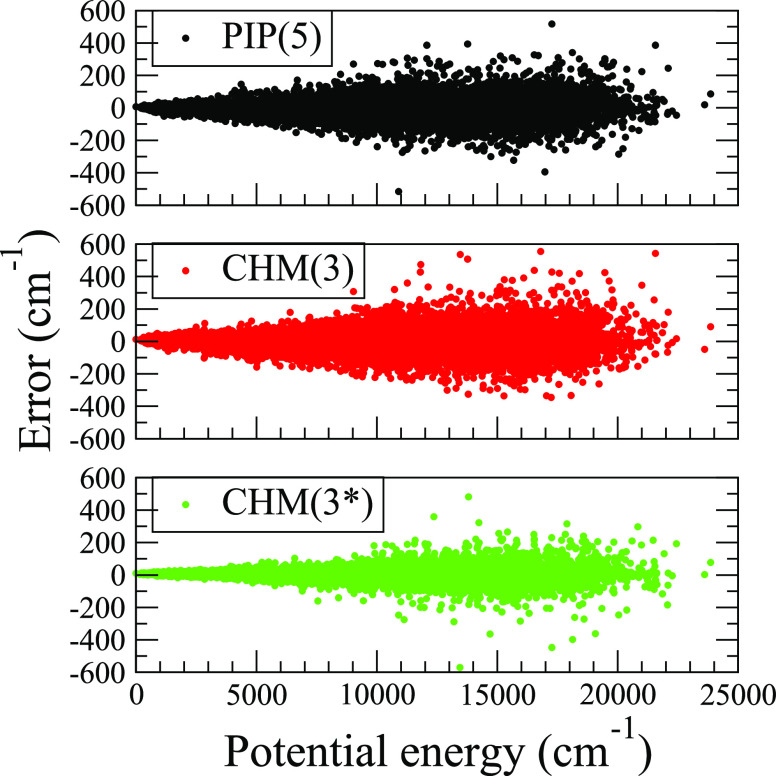
PES test set errors of formamide (H_2_NCHO) fits trained on 12572 configurations and tested on 20852 configurations, calculated at the PNO-LCCSD(T)-F12/cc-pVDZ-F12 level of theory, and shown here for three models: PIP(5), CHM(3), and CHM(3*). The potential energy is relative to the global minimum **MIN1**. The respective training set RMSEs are 55.2, 67.8, and 17.5 cm^–1^, and the corresponding testing set RMSEs are 64.8, 74.0, and 41.8 cm^–1^.

It is informative to make a detailed inspection of the quality of the fits, which we summarize partially in Table 3 and more completely in Figure 6. The data reveal a common pattern of increasing error with the increasing potential energy above the global minimum, with the largest errors occurring near 16,000 cm^–1^, as seen in Figure 6, just below the proton transfer transition state (E(**TS12**) = 16,503 cm^–1^). The large error cluster near that energy is almost entirely due to the high-energy configurations of the two main basins, **MIN1** and **MIN2**, since the RMSE of the **TS12** basin is in fact smaller than the total RMSE despite the high energy of the saddle point. For comparison, the respective test set **TS12** RMSEs of PIP(5), CHM(3), and CHM(3*) are 61, 56 and 24 cm^–1^. Overall, the PIP(5) and CHM(3) errors are very similar, yet CHM(3*) error stays noticeably smaller, particularly for the energies below 10,000 cm^–1^. Additional illustration of CHM(3*) superior performance is found in Table 3, where the energies of individual stationary points are compared for the three models. Particularly interesting is the case of the **MIN5** isomer, a rarely occurring *cis* OH/CH/NH alignment, and the configurations in its vicinity, including the rotational saddle point **TS25** (refer to Figure S5), which incidentally were not sampled in the training set due to a combination of the saddle point’s high energy and a not sufficiently long trajectory propagation. All three fits fail to recover **MIN5/TS25** energies by wide margins; however, both CHM models perform significantly better than PIP(5), with the error decreasing from CHM(3) to CHM(3*). This result is consistent with the findings on the outlier “giraffe” structures in CH_5_^+^, where CHM predicted the test structures much better than the higher-order PIP model.

**Table 3:** Energies (in cm^–1^) of the Key Stationary Points on the Formamide (H_2_NCHO) PES Relative to the Global Minimum **MIN1** along with the Fitted Models’s PIP(*M*) and CHM(*M*) with the Corresponding PIP Basis Sizes *L*, Energy Errors Given in Italics Relative to the Reference Energy Values REF^a^

	**MIN2**	**MIN3**	**MIN4**	**MIN5**	**TS1**	**TS2**	**SP**	**TS12**	**TS34**	**TS25**	**TS23**	**TS45**
REF	4264	5400	5524	6075	6342	6842	8364	16,503	7742	8060	12,603	13,721
PIP(5)	4264	5306	5457	12,560	6295	6772	8035	16,526	7645	11,220	12,758	15,400
*L* = 3292	*0*	*–94*	*–67*	*6485*	*–47*	*–70*	*–329*	*23*	*–97*	*3160*	*155*	*1679*
CHM(3)	4244	5250	5376	11,038	6261	6949	8081	16,525	7674	10367	12519	14,717
*L* = 220	*–20*	*–150*	*–148*	*4963*	*–81*	*107*	*–283*	*22*	*–68*	*2307*	*–84*	*996*
CHM(3*)	4259	5340	5476	10,285	6331	6898	8195	16,508	7719	9675	12674	14,613
*L* = 611	*–5*	*–60*	*–48*	*4210*	*–11*	*56*	*–169*	*5*	*–23*	*1615*	*71*	*892*

a **MIN2**–**MIN5** represent HN=CH–OH, formimidic acid isomers; **TS1**/**TS2** are NH_2_ rotation saddle points; **TS12** is the N–H–O proton transfer transition state; **SP** is an NH_2_-flip second-order saddle point; **TS34**–**TS45** are the bond rotation saddle points connecting **MIN2**–**MIN5**. The data used to train the models and the reference stationary point geometries and energies (REF) are from the B3LYP/aug-cc-pVDZ/GD3 training set described in the text.

## 4 Conclusions and Outlook

We have described an approach to fitting molecular PESs using the Hartree–Fock (HF) expression for the energy, or more specifically, its simplified form that is linearly dependent on the density matrix and is labeled as the core Hamiltonian model (CHM) since only the electron–nucleus interactions are modeled explicitly. Instead of fitting the *ab initio* potential energy directly using a set of PIPs, we effectively decompose the energy (*sans* the trivial nuclear repulsion) into a sum of an individual atom, atom-other-atom, and additionally a standalone correction energy (Δ*E*) contributions and represent them using linear combinations of a single set of PIPs. This approach also bears a formal resemblance to the current state-of-the-art Δ-machine learning approach to PES fitting using PIPs where a low-level (LL) PES, generally constructed with a large PIP basis (*L*-large), is corrected by additional sparse high-level (HL) *ab initio* data, *E*_HL_ – *E*_LL_, that roughly corresponds to the electron correlation energy and is fitted with a small PIP basis (*L*-small) and as a general rule, for high-permutational symmetry systems *L*-large ≫ *L*-small. The key difference is that the CHM model can use as input the small PIP basis (*L*-small) to generate a fit of the same quality.

We have shown that even the most primitive representation of the electron density, i.e., a single *s*-type Gaussian function per atom center, can achieve rather remarkable results in conjunction with a low-order PIP basis. Notably, it has been demonstrated on a series of carefully chosen test molecules (the two-electron one-dimensional HeH^+^ and three-dimensional H_3_^+^ ions, a fragmented methanium ion, CH_5_^+^, and a formamide molecule that undergoes isomerization) that the linearly parameterized, CHM can achieve the same level of fit precision, judged by being tested on independently generated test sets, as the conventional PIP approach with a PIP order of 2 degrees lower than that of the conventional method. The latter result amounts to using approximately 10–20% of the conventional PIP terms and with a little extra computational cost that typically arises from the operational overhead of calculating the multiple energy contributions. This finding is particularly promising for large-scale applications to molecules with high-permutational symmetries for which generating a high or even moderate order PIP is computationally highly problematic, one potential example being the C_20_ fullerene, while both a high quality of electronic structure theory, e.g., an appropriate CCSD(T) level, and a precise fit of the corresponding global PES are desired. As a brief note of caution on large-scale applications, we add that the CHM should work well for closed-shell single-determinant dominated molecules where Δ*E* is much smaller than *E*_HF_ and is a slowly varying function of nuclear configuration, as demonstrated by the present calculations. The cases where CHM in its present formulation may potentially have problems are related to open shell multideterminantal systems, such as radical complexes and complexes involving *d*-elements (transition metals) and heavier elements. Further investigation of these phenomena is required.

As our foremost goal is to demonstrate the new method’s performance to advance the field, the PESs reported here are not designed to be suitable for production calculations, except for exploratory MD simulations or configuration sampling for further PES improvement while also considering that some of them have been published previously, we wish to spell out the following perspectives:

(i) It is evident that a more intelligent selection of PIPs, one that is not based on the total power *M* while naively symmetrizing monomials over all of the factorial permutations, is expected to make the CHM model extremely efficient. In the present work, it has been shown for the H_3_^+^ and the formamide cases that CHM can produce precise trained and test fits using augmented first and third-order PIPs, respectively. In other words, a few (dozens of) well-selected PIPs input into the CHM model can produce high-quality fits. For instance, one of such selection approaches, which is based on PIP term contribution to the RMSE, has been discussed in the literature specifically for applications to generating PESs of large molecules with relatively high-permutational symmetry.^31^
(ii) In the calculations reported here, the atoms are treated as spherical particles. Based on the work of others,^35^ we realize that it is desirable to expand the density using the higher angular momentum orbitals: *p*, *d*, ··· as this produces a better description of *ab initio* data by way of adding higher-order many-body interactions into the HF energy expression. This also adds variational degrees of freedom by means of introducing *sp*, *pp*, *sd*, ··· density matrix elements, potentially leading to a steep decrease of the PIP order needed for a precise fit. All of the presently considered models extended in such a way would, of course, require more extensive and diverse training sets but not significantly so as the present calculations have shown, mainly to avoid problems associated with overfitting, yet the benefits of using low-order PIPs (*M* = 1 or 2) for high permutational symmetry molecular systems are expected to be enormous.
(iii) The possibility of formulating the full HF model with explicit electron–electron (Coulomb and exchange) interactions and upgrading the simple CHM model via the nonlinear dependence of the HF energy on the density matrix is a worthwhile avenue to explore. So far, we have considered approximate two-electron two-center (2e2c) and two-electron three-center (2e3c) contributions using a linear formulation with a non-interacting-atom expression for the density matrix, albeit with no tangible improvement in the fit. An exact HF formulation with a nonlinear regression procedure is expected to yield better results, yet understandably at a much higher computational cost for finding the best variational coefficients **c**.
(iv) As a final perspective, we wish to point out that for large-scale numerical applications, evaluation of the gradients not yet considered at the present stage of development will become crucial. Implementation of the CHM energy (eq 12) derivatives is expected to be as efficient as the energy evaluation due to the fewer PIPs required for the fits, exemplified by the energy evaluation timing tests. The main effort remains differentiation of the PIPs, and to this end, the recently published PESPIP software^64^ and the associated numerical tools should make this task for the CHM model numerically efficient.

## Supporting Information Available

Expressions for the Gaussian matrix elements; details of asymptotic limits of density matrix elements; examination of models beyond the core Hamiltonian model; HeH^+^ energy decomposition in the CHM model; details of CH_5_^+^ data sets and fits; and isomerization landscape and set pruning for H_2_NCHO (PDF)
