# Supplementary material for: Fitting Potential Energy Surfaces by Learning the Charge Density Matrix with Permutationally Invariant Polynomials
Source: J Chem Theory Comput. 2023 Aug 10;19(17):5690–700. doi: 10.1021/acs.jctc.3c00586 (PMC10501011; doi:10.1021/acs.jctc.3c00586)
Supplement: Supplementary file 1 — ct3c00586_si_001.pdf [file ct3c00586_si_001.pdf]

Supplementary Information

Fitting potential energy surfaces by learning the charge density matrix with permutationally invariant polynomials

Younos Hashem[1], Katheryn Foust[1], Martina Kaledin,[1,*] Alexey L. Kaledin[2,*]
[1] *Department of Chemistry & Biochemistry, Kennesaw State University, 370 Paulding Ave NW, Box # 1203, Kennesaw, Georgia, 30144*
[2] *Cherry L. Emerson Center for Scientific Computation and Department of Chemistry, Emory University, 1515 Dickey Drive, Atlanta, Georgia, 30322*

Table of Contents

S1. Gaussian matrix elements......2

S2. Asymptotic limits of density matrix elements......3

S3. Beyond the core Hamiltonian model......3

S4. HeH^+ energy decomposition in the CHM model and H_3^+ fitting details......5

S5. Details of CH_5^+ data sets and fits......8

S6. Isomerization landscape and set pruning for H_2NCHO......12

S1. Gaussian matrix elements

This section provides analytic expressions for the Gaussian integrals seen in Eqs. 2-5 of the main text used in the present calculations. Since we limited our model to the *s*-density with a single function per atomic center, only the integrals involving *s* functions will be given explicitly. The normalized *s* function on center *A* with coordinate $\mathbf{R}_A$ is

$$\phi_A(\mathbf{r}) = \left(\frac{2\alpha_A}{\pi}\right)^{\frac{3}{4}} exp[-\alpha_A|\mathbf{r} - \mathbf{R}_A|^2] \qquad (S1)$$

where **r** is electron's coordinate. The kinetic energy integral is

$$T_{AB} = \frac{\alpha_A\alpha_B}{\alpha_A + \alpha_B}\left(3 - 2\frac{\alpha_A\alpha_B}{\alpha_A + \alpha_B}R_{AB}^2\right)S_{AB} \qquad (S2)$$

where the overlap integral is

$$S_{AB} = \left(\frac{4\alpha_A\alpha_B}{(\alpha_A + \alpha_B)^2}\right)^{\frac{3}{4}} exp\left[-\frac{\alpha_A\alpha_B}{\alpha_A + \alpha_B}R_{AB}^2\right]. \qquad (S3)$$

The distance between two centers *A* and *B* is $R_{AB} = |\mathbf{R}_A - \mathbf{R}_B|$. The one-electron Coulomb integral involving two basis functions centered at *A* and *B* nuclear centers coupled over the nuclear center *C* with charge Z_C is

$$V_{ABC} = S_{AB}Z_C\frac{erf[\sqrt{\alpha_A + \alpha_B}R_{1C}]}{R_{1C}} \qquad (S4)$$

where the centroid position vector is $\mathbf{R}_1 = (\mathbf{R}_A\alpha_A + \mathbf{R}_B\alpha_B)/(\alpha_A + \alpha_B)$. For *A* = *B*, the above integral reduces to

$$V_{AAC} = Z_C\frac{erf\left[\sqrt{2\alpha_A}R_{AC}\right]}{R_{AC}} \qquad (S5)$$

and for *A* = *B* = *C* it reduces to

$$V_{AAA} = 2Z_A\left(\frac{2\alpha_A}{\pi}\right)^{\frac{1}{2}} \qquad (S6)$$

Finally, the 2-electron integral over four centers *A*, *B*, *C* and *D* is

$$I_{CD}^{AB} = S_{AB} S_{CD} \frac{erf\left[\sqrt{\lambda} R_{12}\right]}{R_{12}} \qquad (S7)$$

where $\lambda = \frac{(\alpha_A+\alpha_B)(\alpha_C+\alpha_D)}{\alpha_A+\alpha_B+\alpha_C+\alpha_D}$ and $R_{12} = |\mathbf{R}_1 - \mathbf{R}_2|$ with $\mathbf{R}_2 = (\mathbf{R}_C\alpha_C + \mathbf{R}_D\alpha_D)/(\alpha_C + \alpha_D)$. One can reduce Eq. S7 to all the special cases by the standard analysis.

S2. Asymptotic limits of density matrix elements

The scaling function that appears in Eq. 8 (main text) is defined as a sum of products of long- and short-range one-dimensional damping functions that act on the non-constant part of the density element P_{aa},

$$\sigma_a = \sum_{b \neq a} exp[-r_{ab}/d_0]\, exp[-d_0/r_{ab}] \qquad (S8)$$

The long-range damping with $r_{ab} \to \infty$ for all *b* while $\sigma_a \to 0$ makes sure that an isolated atom *a* has exactly n_a electrons. The short-range damping is introduced mainly to control blow-up in the P_{aa} behavior at short r_{ab} distances but may additionally be thought of as an enforcement condition for wavefunction collapse in the united atom limit. Namely, for a diatomic molecule *ab* the following must hold for $r_{ab} = 0$ regardless of the linear coefficients c_i^{atom} and c_i^{pair},

$$P_a(r_{ab}) + P_b(r_{ab}) + 2P_{ab}(r_{ab})S_{ab}(r_{ab}) = n_a + n_b \qquad (S9)$$

which is guaranteed by Eqs. 8, 10 in the main text, and Eq. S8.

S3. Beyond the core Hamiltonian model

The exact Hartree-Fock energy is given in Eq. 7 (main text) and is reproduced here for clarity but with an altered notation,

$$E_{\mathrm{HF}} = \sum_{ab} P_{ab} \left(T_{ab} + V_{ab} + \frac{1}{2} \sum_{cd} Q_{cd} \left[I_{cd}^{ab} - \frac{1}{2} I_{cb}^{ad} \right] \right) + E_{\mathrm{nuc}} \qquad (S10)$$

where we have explicitly distinguished the density matrix appearing in the two-electron contribution to the energy: Q_{cd}. With $\mathbf{Q} = \mathbf{P}$ the equation is the true HF energy. However, for a linear class of least squares solutions of the unknown coefficients c_i^{atom} and c_i^{pair}, we cannot express $\mathbf{Q}$ in terms of these coefficients but approximate it using some reasonable models. Here we will examine two such models based on the following approximation,

$$\begin{cases} Q_{cc} = n_c \\ Q_{cd} = 0 \end{cases} \qquad (S11)$$

Although it is a rough approximation, it is symmetric and precisely conserves the overall charge. Additionally, it assumes that there can be no more than three-center integrals, leading to the two-electron/two-center model by retaining only I_{cc}^{aa} and I_{ca}^{ac} integrals (2e2c), and the full two-electron/three-center model (2e3c).

In Table S1, we review calculations based on these two models along with the Core Hamiltonian Model (CHM) for H_3^+. As can be seen, the 2-electron/3-center models do not perform better than the CHM for a range of selected PIP basis sets. However, it is possible to detect a convergence of the error to the same limit with an increasing PIP basis. This leads to two main conclusions, (*i*) the present linear approximation given by Eq. S11 is not useful and in fact, impractical given the need to calculate the two- and three-center integrals, and (*ii*) to take full advantage of the exact HF energy expression, it may be necessary to solve for the linear coefficients c_i^{atom} and c_i^{pair} using non-linear regression methods. The latter point is being considered in our ongoing investigations.

Table S1. Comparison of trained RMSE (cm^{-1}) of the CHM model and two-electron models involving 2-center (2e2c) and 3-center (2e3c) integrals using H_3^+ fits on the training set generated with a CCSD/cc-pVDZ trajectory propagated at a 7335 cm^{-1} total energy using 3000 points. The PIP orders are M = 1; M = 1 augmented with y_{ij}^2 terms (M = 1*); M = 1 augmented with y_{ij}^2 and y_{ij}^3 terms (M = 1**); M = 1 augmented with y_{ij}^2, y_{ij}^3 and y_{ij}^4 terms (M = 1***); and M = 2.

	CHM(*M*)	CHM+2e2c(*M*)	CHM+2e3c(*M*)
M = 1	92.5	120.2	136.9
M = 1*	15.2	15.4	14.8
M = 1**	2.3	3.7	4.2
M = 1***	1.6	2.1	2.2
M = 2	2.8	2.7	3.0

S4. HeH^+ energy decomposition in the CHM model and H_3^+ fitting details

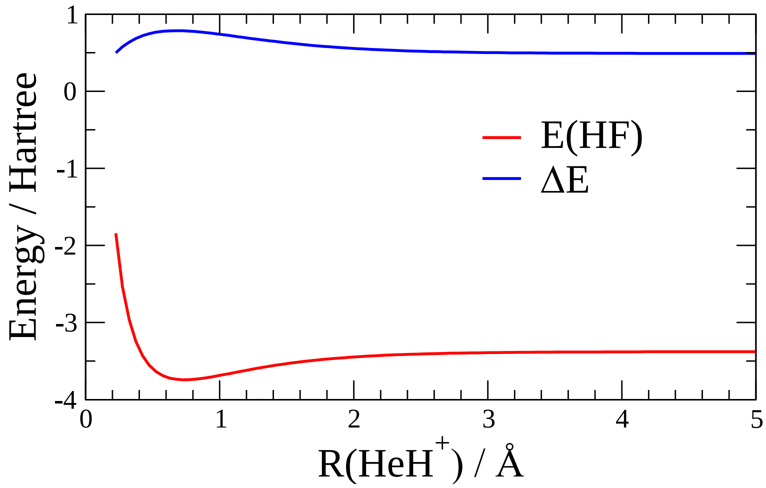

Figure S1. Energy decomposition of the CHM(4) model for the HeH^+ potential energy curve calculated at CCSD/cc-pVDZ level of theory. E(HF) is the component of the Core Hamiltonian energy with the nuclear repulsion energy, and ΔE is the conventional PIP based correction as seen in Eq. 12 (main text).

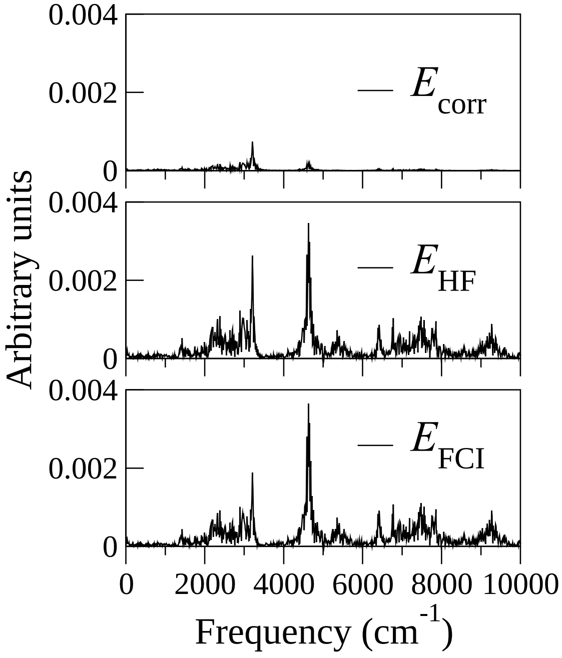

Figure S2. Fourier transforms of the potential energy elements of H_3^+ as functions of time of a classically propagated trajectory. The propagation was done as an NVE ensemble with 3000 steps and a time step of 1 fs with the total energy of 7335 cm^{-1}, roughly corresponding to the harmonic zero-point vibrational energy, ZPVE, and one quantum of the asymmetric stretch. E_{corr} is the correlation energy, E_{HF} is the Hartree-Fock energy, and E_{FCI} is the CCSD/cc-pVDZ energy equivalent to full CI.

In Figure S2, we observe the contribution of E_{corr} to E_{FCI}, where the former is only a small fraction of the latter. The low frequency contributions of the vibrational spectrum almost completely describe the correlation energy. Furthermore, the main correction occurs at the low end of the frequency spectrum, suggesting a slowly varying function. Note the frequency maps into double-frequency for a standard normal mode spectrum since the potential energy is proportional to coordinate-squared in the harmonic approximation.

Table S2. Training set RMSEs (cm^{-1}) of the HeH^+ and H_3^+ fits of their potential energies calculated at the CCSD/cc-pVDZ level of theory. 100 and 3000 points were used to train the HeH^+ and H_3^+ fits, respectively. The PIP orders are M = 1; M = 1 augmented with y_{ij}^2 terms (M = 1*); M = 1 augmented with y_{ij}^2 and y_{ij}^3 terms (M = 1**); M = 1 augmented with y_{ij}^2, y_{ij}^3 and y_{ij}^4 terms (M = 1***); and M = 2. The corresponding PIP basis size L is also given.

	HeH^+				H_3^+			
	L	PIP(*M*)	*L*	CHM(*M*)	*L*	PIP(*M*)	*L*	CHM(*M*)
M = 1	2	10272	5	875	2	1920	4	92
M = 1*					3	118	7	15
M = 1**					4	103	10	2
M = 1***					5	103	13	1.6
M = 2	3	2195	9	98	4	119	10	2.8
M = 3	4	1825	13	7.2	7	27	19	0.4
M = 4	5	630	17	1.5	11	1.7	31	0.1
M = 5	6	174			16	0.5		
M = 6	7	109						
M = 7	8	28						
M = 8	9	12						

S5. Details of CH_5^+ data sets and fits

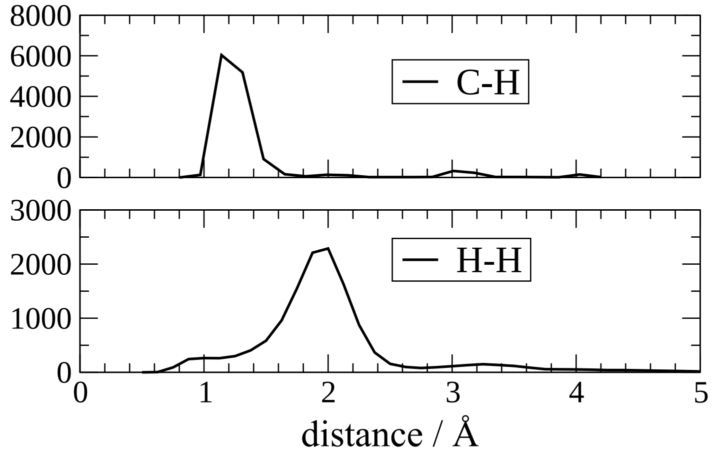

Figure S3. Pair distribution functions of CH_5^+ training set of 13605 configurations calculated at the B3LYP/cc-pVTZ level of theory.

Table S3. Training and testing RMSEs (in cm^{-1}) of CH_5^+ fit trained on 13605 configurations and tested on 52400 configurations, calculated at the B3LYP/cc-pVTZ level of theory.

	training set		testing set	
M	PIP(*M*)	CHM(*M*)	PIP(*M*)	CHM(*M*)
1	4133	1847	4090	1827
2	1751	742	1724	730
3	904	329	894	327
4	424	115	409	119
5	159	58	162	68
6	73		87	

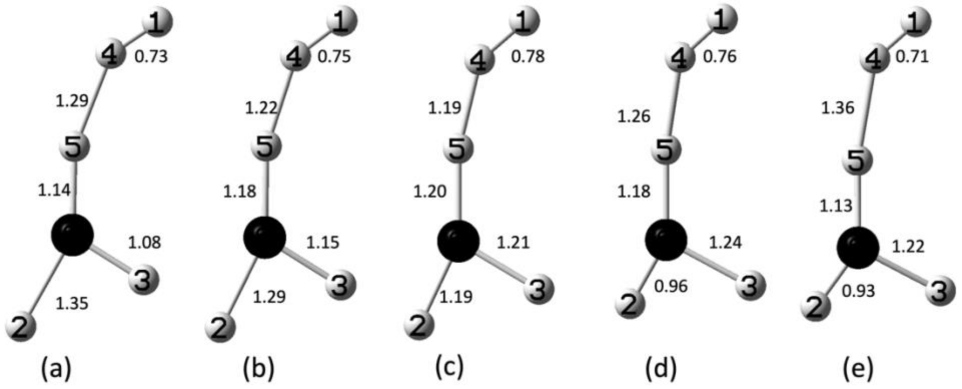

Figure S4. Five outlier "giraffe" configurations of CH_5^+ that appear in the test set but are largely missing from the train set. Only configuration (d) can be loosely identified in the training set. The accompanying numbers are bond distances in Å.

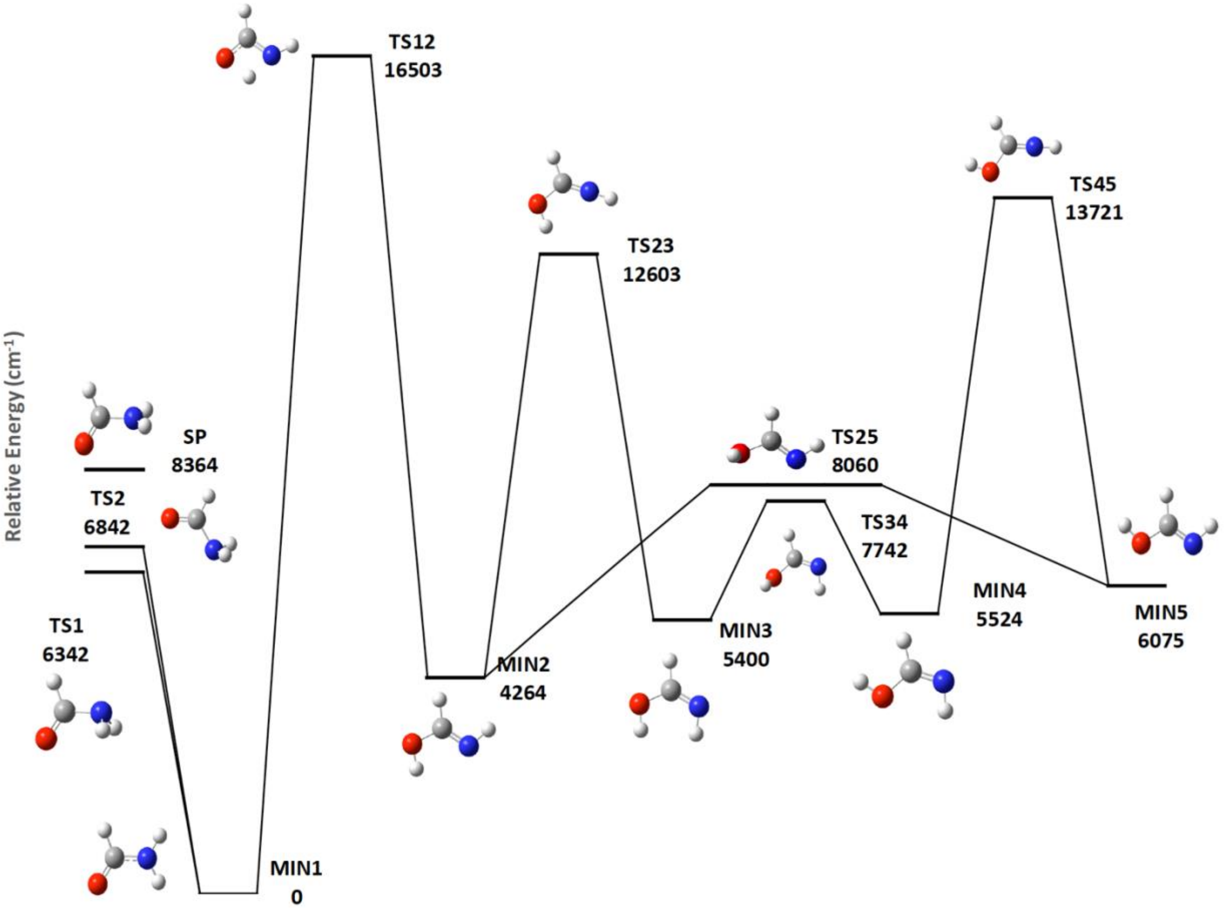

Figure S5. Key stationary points on the formamide and its H-transfer isomer, formimidic acid surfaces provide a representation of the training and testing sets for the reported fits. The numbers next to each structure are electronic energies in cm^{-1} relative to **MIN1** and calculated at the level of B3LYP/aug-cc-pVDZ/GD3. Structures **MIN5** and **TS25** are largely absent from both sets due to an insufficiently extensive sampling.

Table S4: Relative total electronic energies, E_0, zero-point corrected values, E_0+ZPE, and thermal corrections of the Gibbs free energy, E_0+G, respectively in cm^{-1}, kcal/mol for various formamide isomers calculated at B3LYP/aug-cc-pVDZ/GD3 level of theory.

Stationary points	E_0 (cm^{-1})	E_0 (kcal/mol)	E_0+ZPE (cm^{-1})	E_0+ZPE (kcal/mol)	E_0+G (cm^{-1})	E_0+G (kcal/mol)
MIN1	0	0.00	0	0.00	0	0.00
MIN2	4264	12.19	4404	12.59	4463	12.76
MIN3	5400	15.44	5486	15.69	5528	15.81
MIN4	5524	15.79	5566	15.91	5600	16.01
MIN5	6075	17.37	6124	17.51	6170	17.64
TS1	6342	18.13	6085	17.40	6138	17.55
TS2	6842	19.56	6534	18.68	6595	18.86
TS12	16503	47.18	15405	44.04	15473	44.24
TS23	12603	36.03	12053	34.46	12060	34.48
TS34	7742	22.14	7513	21.48	7569	21.64
TS45	13721	39.23	13110	37.48	13129	37.54
TS25	8060	23.04	7823	22.37	7892	22.56
SP	8364	23.91	7802	22.31	7866	22.49

S6. Isomerization landscape and set pruning for H_2NCHO

For a set of trial potential energy distributions $\{f_i(V)\}$ derived from microcanonical trajectories of different total energies numbered $i = 1, 2, \ldots$ each of length N time steps, we seek such coefficients in the "pruned" distribution

$$g(V) = \sum_i c_i f_i(V) \qquad (S12)$$

that

$$\frac{\partial}{\partial \mathbf{c}} \int_0^{V_{max}} dV \left(g(V) - \alpha \frac{M}{V_{max}} \right)^2 = 0 \qquad (S13)$$

where α is a constant to be determined by the $g(V)$ normalization requirement and where M (<< N) is the size of the pruned set.

$$\alpha = \frac{V_{max}}{N^2 \sum_{ij} [\mathbf{S}^{-1}]_{ij}} \qquad (S14)$$

where the overlap matrix is calculated as

$$S_{ij} = \int_0^{V_{max}} dV f_i(V) f_j(V) \qquad (S15)$$

using quadrature. The condition $S13$ ensures that $g(V)$ is as close to a uniform, or a top-hat, distribution as possible by assigning approximately equal weights to the configurations with the potential energies in $[0, V_{max}]$. Carrying out differentiation, the solution for the best coefficients is

$$c_i = \frac{M}{N} \frac{\sum_j [\mathbf{S}^{-1}]_{ij}}{\sum_{ij} [\mathbf{S}^{-1}]_{ij}} \qquad (S16)$$

For practical purposes, we need the frequency of pruned points (sampling intervals) in each trial set i. This frequency is given by the reciprocal of the coefficient $\nu_i = c_i^{-1}$.

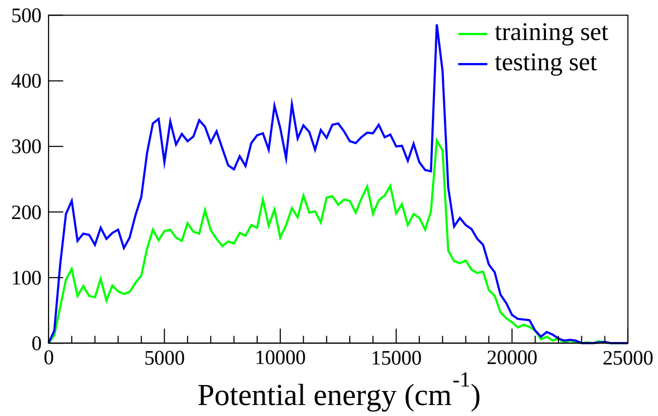

Figure S6. Potential energy distributions of the training and testing sets of H_2NCHO calculated using the PNO-LCCSD(T)-F12 with cc-pVDZ-F12 orbital basis set. The spike at 17000 cm^{-1} is a cluster of configurations describing the vicinity of the proton transfer barrier (**TS12**).

Table S5. Training set RMSE (in cm^{-1}) of H_2NCHO fits for the two electronic structure levels considered in the present work: "B3LYP" = B3LYP/aug-cc-pVDZ/GD3 and "CC" = PNO-LCCSD(T)-F12/cc-pVDZ-F12. Several PIP orders *M* were considered, where 2* and 3* are the augmented sets (see main text for definitions).

	B3LYP		CC	
M	PIP(*M*)	CHM(*M*)	PIP(*M*)	CHM(*M*)
1	4034.5	1313.3	4108.9	1313.4
2	2113.6	374.5	2104.6	381.9
2*	1209.6	156.9	1218.8	160.7
3	947.7	65.9	962.8	67.8
3*	369.1	16.1	375.6	17.5
4	279.5		286.5	
5	51.4		55.2	
